# The Influence of Storage on Human Milk Lipidome Stability for Lipidomic Studies

**DOI:** 10.1021/acs.jproteome.1c00760

**Published:** 2021-12-29

**Authors:** Dorota Garwolińska, Michał Młynarczyk, Agata Kot-Wasik, Weronika Hewelt-Belka

**Affiliations:** Department of Analytical Chemistry, Faculty of Chemistry, Gdańsk University of Technology, Gabriela Narutowicza 11/12, 80-233 Gdańsk, Poland

**Keywords:** human milk, storage, lipidome stability, LC−MS lipidomics

## Abstract

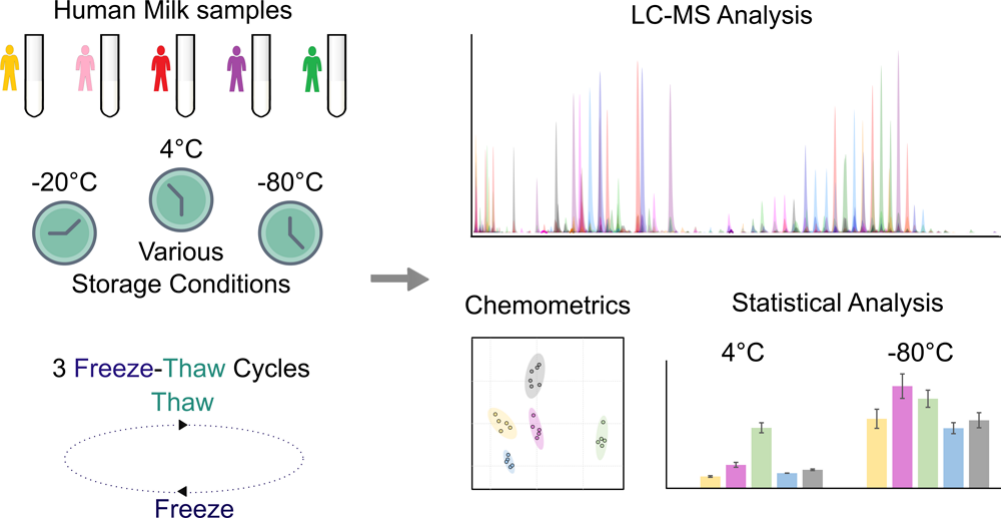

Human milk (HM) lipidome stability during storage is crucial in lipidomic studies to avoid misinterpretations. Facing the lack of comprehensive work on the HM lipidome stability, we performed a study on a potential alteration in the lipid profiles of HM samples stored under different conditions. An untargeted LC-Q-TOF-MS-based approach was applied to study the influence of storage conditions as well as the interaction of the storage temperature and time on HM lipid profiles. The samples were stored for 4–84 days at temperatures in the range from 4 to −80 °C and also were exposed to up to three freeze–thaw cycles. The results showed that the storage at 4 °C for just 4 days as well as being subjected to three freeze–thaw cycles can lead to a change in the content of lipids. The observed differences in levels of some lipid species in samples stored at −20 °C in comparison to the concentration level of those lipids in samples stored at −80 °C were not statistically significant, and inter-individual variance regardless of sample storage condition was maintained. The storage of HM samples at −20 °C for up to 3 weeks and −80 °C for up to 12 weeks ensures sample lipidome stability.

## Introduction

Lipidomics is defined as the field of “omics” research focused on the comprehensive and quantitative analysis of lipids in biological materials. In a lipidomic study, it is expected that observed differences in lipidome reflect real biological changes and do not originate from technical variability. This corresponds also to the HM lipidome studies, when the HM lipid composition is monitored to reveal changes in the course of lactation, between individual mothers or as a response to diet.^1−3^ However, the detected variation may originate not from inter- or intra-individual differences (e.g., determined by maternal diet) but from quality assurance failure in the lipidomic study. This can lead to erroneous biological interpretations and false conclusions.

Lipidomic study design should include the control and minimization of unwanted variation in sample composition to prevent any errors in data interpretation. One of the major sources of error in lipidomic studies is an inappropriate pre-analytical process including sample collection and handling (storage time and condition and freeze–thawing) that may produce degradation or conversion of lipids. Artificial variation in lipid concentration can lead to incorrect interpretation of the data obtained from lipidomic experiments. In an LC–MS-based lipidomic study, the best practice is to analyze the complete sample set in a single batch. Due to that, typically, biological samples are collected and kept in a freezer for various periods ranging from several days to even a few months before analysis. Nevertheless, such practice is appropriate only if lipids contained in the analyzed samples are stable and thus no alteration in lipid concentration occurs during storage.

The importance of the pre-analytical aspects for the lipidomic study output resulted in the establishment of the collection and handling protocols for biofluids such as blood and urine.^4−8^ However, it is not available for HM samples. A previous research that studied the effects of storage on HM components focused mainly on HM macronutrient content. The lipid fraction was analyzed as total fat (corresponds to the total lipid-soluble fraction of the sample including all HM lipids) or the total amount of lipid classes.^9−12^ In only one research, besides total fat analysis, the content of individual unesterified fatty acids was determined.^13^

To follow up potential influence of sample storage, the stability of lipid profiles in HM samples, which were stored for different periods and under various temperature conditions, were investigated using untargeted lipidomics. HM samples were collected from five healthy female volunteers, divided for storage under different conditions, and further analyzed by reversed-phase liquid chromatography coupled with mass spectrometry (LC-Q-TOF-MS) at intervals. The sets of samples were stored under different conditions. Three additional sets of the samples underwent a series of up to three freeze–thaw cycles. We applied an analytical approach that allowed simultaneous detection of high-abundance glycerolipids and low-abundance phospholipids in one LC–MS run. The comparative analyses were performed with the use of chemometrics and statistical tools in terms of lipid composition alterations among several lipid classes depending on storage conditions.

## Experimental Section

### Ethical Approval

Research ethics approval was obtained from the Human Research Ethics Committee of the Medical University of Gdańsk, Poland (decision no. NKBBN/389/2019, date of approval: 8th of July 2019).

### Reagents and Materials

Reagents and chemicals used in this study are described in the Supporting Information.

### HM Sample Collection and Storage

HM samples were donated by five healthy female volunteers being at different lactation periods. The HM samples were collected by the full expression of one breast using an electronic breast pump at approximately 10 a.m. After collection and gentle shaking, approximately 10 mL of HM was transferred into sterile disposable tubes and delivered to the laboratory (in the cool box with the temperature below 4 °C, samples were not frozen). The time between the collection, sample aliquoting, and placing in the freezer constituted approximately 4 h. Written informed consent was obtained from each participant. The characteristic of collected samples is presented in Table 1.

**Table 1:** Characteristics of the Analyzed Milk Samples

no.	sample ID	woman ID	month of lactation
1	13A_1, 13A_2	W13A	13
2	8A, 8B	W8	8
3	18A, 18B	W18	18
4	13B_1, 13B_2	W13B	13
5	4A, 4B	W4	4

A quality control (QC) sample was immediately prepared by pooling equal volumes of 2 mL from each of the five collected HM samples. Afterward, sets of 750 μL aliquots were prepared for each HM sample and the QC and extraction blank sample (deionized water instead of biological material) into sterile Eppendorf tubes. Each HM sample and blank extraction sample was aliquoted into 11 separate tubes, while the QC sample was aliquoted into 17 separate Eppendorf tubes. In consequence, 10 sets of samples consisting of 6 HM samples, QC sample, and blank extraction sample were obtained.

The sets of samples were stored under different conditions. Concerning storage temperatures, the samples were exposed to +4, −20, and −80 °C. Concerning the storage duration, the samples were stored at +4 °C for 4 days; at −20 °C for 4, 7, and 14 days; and −80 °C for 7, 21, and 84 days. To study the influence of freezing and thawing on the stability of lipidome of HM samples, three sample sets underwent a series of three freeze–thaw cycles. All sample sets were frozen at −80 °C. On the first day, each set was left to thaw for 1.5 h at room temperature. Then, after thorough mixing, two sets were returned to the freezer and one was analyzed. On the second day, two sample sets were left to thaw for 1.5 h and thoroughly mixed in a vortex mixer. Afterward, one set was returned to the freezer and one was analyzed. On the third day, the last sample set was left to thaw for 1.5 h, thoroughly vortexed, and analyzed. Consequently, three sample sets have undergone from one to three freeze–thaw cycles.

### Sample Preparation

HM samples were thawed at room temperature before extraction. Extraction of lipids was based on a previously published dilution-enrichment LLE- and SPE-based strategy with small modifications.^14^ Details are described in the Supporting Information.

### LC–MS Analysis

HM metabolite profiling was performed with an LC-Q-TOF-MS system: an Agilent 1290 LC system equipped with a binary pump, an online degasser, an autosampler, and a thermostated column compartment coupled to a 6540 Q-TOF-MS with a dual electrospray ionization (ESI) source (Agilent Technologies, Santa Clara, CA, USA). To monitor global HM lipidome, reversed-phase chromatography has been implemented. The chromatographic and mass spectrometry condition was published previously.^14^ Details are described in the Supporting Information. A volume of 10 μL was taken from each lipid extract and pooled to form the quality assurance (QA) sample, which served to assess the analytical acquisition quality within a batch.^15^ HM samples were injected randomly, and QC samples were injected every five injections to control LC-Q-TOF-MS stability and to assess data quality. Also, PC 18:0/18:0 was added to the final extracts (1 μg/mL) and was used as an internal standard to control the stability of the LC–MS system.

### Data Treatment

The peak areas of the identified human milk lipids from the raw LC–MS data were obtained using the Batch Targeted Feature Extraction algorithm implemented in the Agilent MassHunter Workstation Profinder 10.0 (Agilent Technologies, Santa Clara, CA, USA). As an input, self-prepared lipid database containing molecular formulas, monoisotopic masses, and retention time information of HM lipids was used. Lipids included in the database were identified by comparing the mass accuracy of potential lipids against the custom database (Δ5ppm tolerance) and manual interpretation of the obtained MS/MS spectra of HM samples.^3^ Details regarding feature extraction, data alignment, and filtration are included in the Supporting Information. Only molecular features (MFs) that fulfilled the criteria of frequency (MFs detected in all samples that were stored under the specified conditions) and MF’s volume %RSD (<30%) in all analyzed QC samples (3 extraction replicates) were included in further statistical and chemometric analysis. Finally, peak volumes of 28 TGs, 15 DGs, 4 lysoPCs, 2 lysoPEs, 4 PCs, 4 PEs, and 6 SMs were included in the calculation. Statistical tests, %RSD, and fold change calculations were conducted using the peak volume or percentage relative amount of lipids within the specified lipid class (calculated in Microsoft Excel 2016 software (Microsoft Corporation, Redmond, WA, USA) by dividing the lipid species peak volume by the sum of the peak volume of all lipid species detected within the specific class). The statistical analyses (ANOVA unequal variance test) and chemometric analyses including PCA were conducted using the Metaboanalyst online package (http://www.metaboanalyst.ca/). The data for the analysis in Metaboanalyst 4.0 were prepared in Microsoft Excel 2016 software (Microsoft Corporation, Redmond, WA, USA).

## Results and Discussion

In this study, we investigated the influence of sample storage conditions (temperature, duration, and freeze–thaw cycles) on the stability of lipidome in HM samples employing an untargeted lipidomic approach. We focused on the main components of the HM lipid fraction: phospholipids (lysoPCs, lysoPEs, PCs, PEs, and SMs) and TGs. The DGs that originate majorly from the TG lipolysis performed by human milk lipoprotein lipases present in raw milk were also included in the analysis.^16−20^

**Figure 1:**
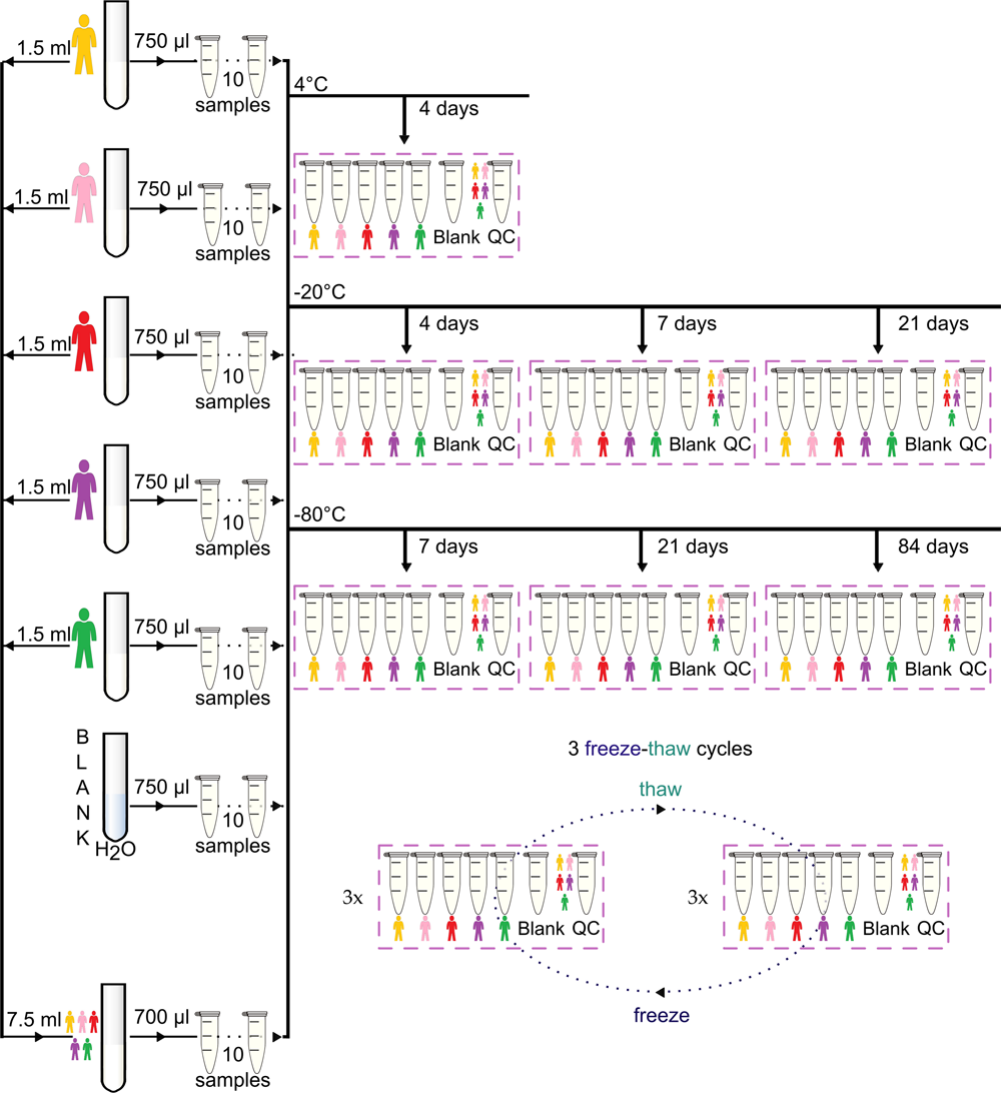
Schematic of the experimental design.

We studied the lipidome stability of samples stored at +4 °C for 4 days; at −20 °C for 4, 7, and 14 days; and at −80 °C for 7, 21, and 84 days. While it is often not possible to analyze the HM samples on the day of collection and typically samples are frozen immediately after collection, samples stored at −80 °C were used as a baseline reference in this study. Reports regarding metabolome stability studies of other biological samples had previously shown that this temperature enables one to maintain the stability of metabolome.^4,8,21^ The integral experimental design is detailed in Figure 1.

We compared the lipid profiles of the studied samples stored under different conditions and evaluated variation of the lipid contents by the calculation of (i) the relative standard deviation (%RSD) of the volumes of MFs detected under a specific temperature condition (−20 vs −80 °C) and (ii) the fold change of the content of lipid species between a group of samples stored under different temperatures (the fold change value correspond to the average peak volume of lipid species in one group of samples over another). The inter-individual variance regardless of sample storage condition was investigated using PCA visualization and statistical tests.

### Glycerolipids

Finding an appropriate method of data normalization for glycerolipids (both diacylglycerols and triacylglycerols) was complicated due to the great diversity of lipid concentration levels detected in samples stored under 4 °C and other temperatures. Comparison of % relative amounts (calculated by the division of a peak volume of a lipid species by the sum of the peak volumes of all lipids within the specific class) of a given lipid caused falsification of lipidomic variances because of the significant decrease and increase in content of a few TG and DG, respectively, with simultaneous lack of or only small changes in the concentration level of other TG and DG in samples stored at 4 °C. Due to that, comparison of lipid profiles of samples stored in 4 and −80 °C was performed on peak volumes instead of relative amounts. To avoid bias in the data arising from LC-Q-TOF-MS signal batch intensity differences, only samples with similar peak areas of the internal standard (%RSD < 15%) were included (first 3 weeks of the experiment) in this part of the data analysis.

First, we studied the stability of the TGs contained in the samples collected from the individual women (*n* = 5) and stored separately at −20 °C (W4, *n* = 5; W8, *n* = 6; W13A = 6; W13B, *n* = 6; W18, *n* = 6) and −80 °C (W4, *n* = 6; W8, *n* = 5; W13A, *n* = 5; W13B, *n* = 5; W18 *n* = 5) during different durations by the calculation of the relative standard deviation (%RSD) of the MF volumes. The results showed that all of the detected TGs (*n* = 28) had acceptable %RSD values among all samples stored at −20 and −80 °C (in the case of −20 and −80 °C, it was for 7, 14, and 21 days and 7, 21, and 84 days, respectively) among all women (below 20% in the case of samples stored at −80 °C and below 30% in the case of samples stored at −20 °C). Due to that, in subsequent data analysis, samples stored under specific temperature conditions for different storage times were considered together as one group of samples. The calculated %RSD values are presented in Table S1.

**Figure 2:**
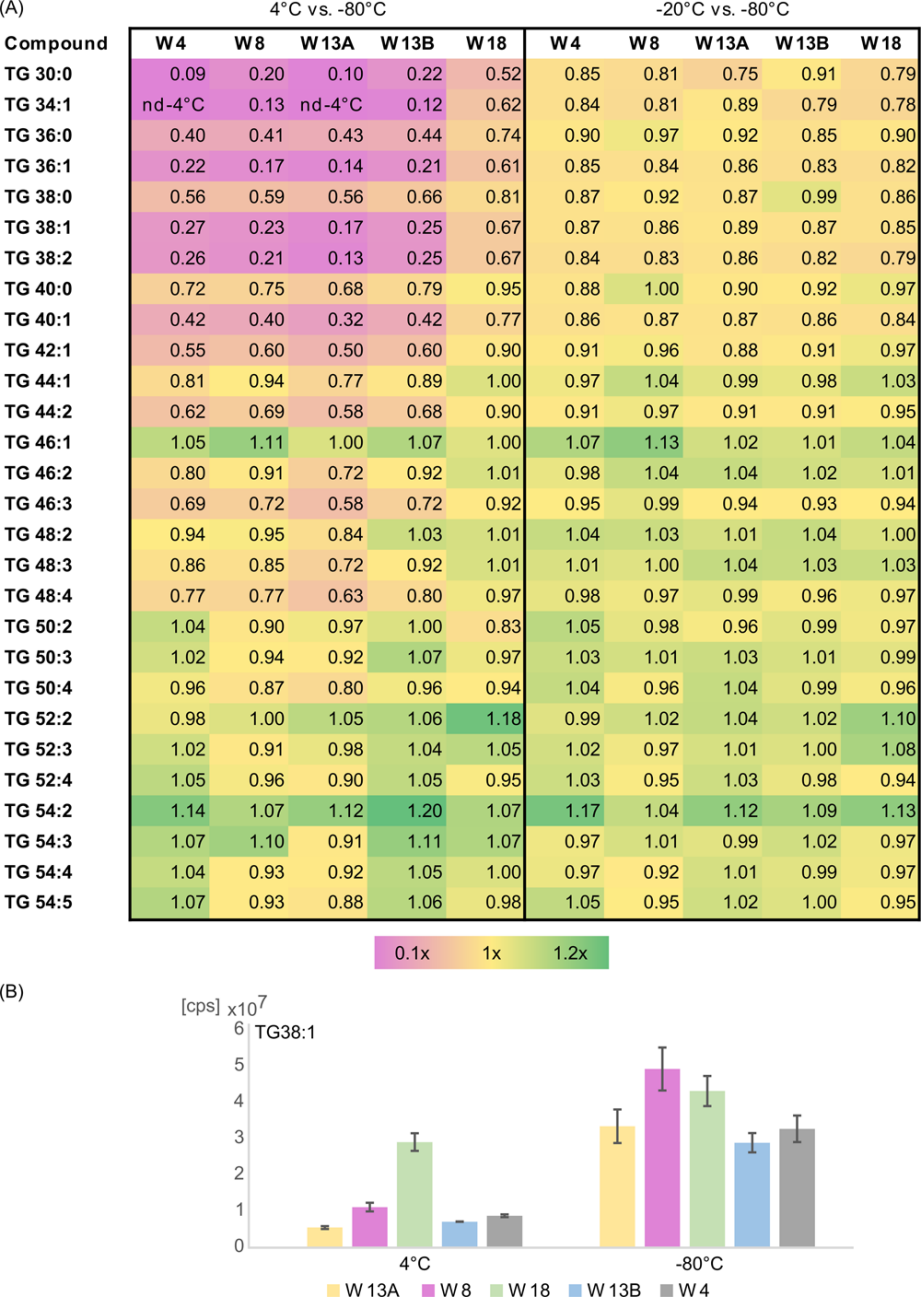
(A) The fold changes of TGs species peak volumes are visualized as a heat map showing the levels of content increase (green, fold change > 1) and decrease (purple, fold change < 1) between tested storage conditions: 4 vs −80 (five left panels) and −20 vs −80 (five right panels). Average fold change was calculated for TG species in samples donated by all women and stored at −20 and −80 °C. (B) Average peak volumes of exemplary TG (TG 38:1) detected in samples collected from particular women and stored under studied temperature conditions (4 °C (*n* = 2) and −80 °C (*n* = 4); error bars represent the standard deviation (SD) of a data set).

To further study the difference between the samples stored at −20 and −80 °C, we performed a statistical test (Mann–Whitney unpaired test). The % relative amount of TG species in samples stored at −20 and −80 °C was similar, all TGs included in the test (*n* = 28) were not statistically significantly changed (*p* < 0.05) with a fold change below 25% (except TG34:1 (*p* < 0.005), TG40:1 (*p* < 0.005), and TG44:2 (*p* < 0.005) that were indicated as significantly changed among samples donated by W18). The fold changes of TG species peak volumes are visualized as a heat map in Figure 2A.

In the next step, the stability of the TGs contained in the samples collected from the individual women (*n* = 5) and stored at 4 °C (W4, *n* = 2; W8, *n* = 2; W13A, *n* = 2; W13B, *n* = 2; W18 *n* = 2) and −80 °C (W4, *n* = 4; W8, *n* = 4; W13A, *n* = 4; W13B, *n* = 4; W18 *n* = 4) was investigated. The calculation of the average fold change of the MF peak volumes indicated a significant decrease in the concentration level of many TGs in samples stored at 4 °C in comparison to samples stored at −80 °C. Importantly, the value of calculated fold change was at a different level in various women, as shown in Figure 2A,B.

**Figure 3:**
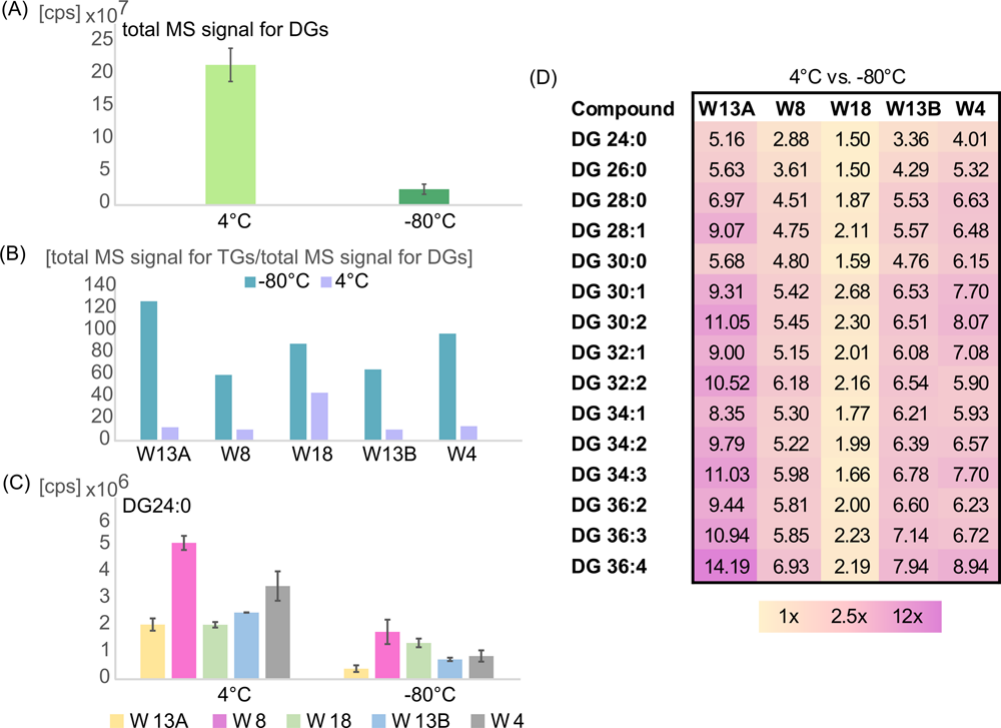
(A) Average total MS signal of DGs detected in HM samples collected from W13A and stored at 4 °C (*n* = 2) and −80 °C (*n* = 4); error bars represent the SD of a data set. (B) Ratio of the total MS signal for TGs to the total MS signal for DGs in samples stored at −80 °C and in samples stored at 4 °C. (C) Average peak volumes of DG24:0 detected in samples collected from particular women and stored under studied temperature conditions (at 4 °C (*n* = 2) and −80 °C (*n* = 4); error bars represent the SD of a data set). (D) Clustering shown as a heat map. Average fold change was calculated for DG species in samples donated by all women and stored at −20 and −80 °C.

The comparison of the DG’s data revealed the differences in the total MS signal of DGs (sum of peak volume of all DG species) in samples stored at 4 °C in comparison to the total DG’s MS signal in samples stored at −80 °C. The total MS signal of DGs detected in HM samples stored at 4 °C was higher (for each woman at a different degree) than the total MS signal for DGs of HM samples stored at −80 °C, as shown in Figure 3A (example data obtained for samples donated by W13A, where the total MS signal for DGs of HM samples stored at 4 °C was approximately nine times higher than the total MS signal for DGs of HM samples stored at −80 °C). Moreover, the ratio of the total MS signal for TGs to the total MS signal for DGs in samples stored at −80 °C was higher than in samples stored at 4 °C (Figure 3B). The comparison of peak volumes of MFs detected in samples stored at 4 and 80 °C exposed a higher content of lipids in samples stored at 4 °C (Figure 3C) than in samples stored at −80 °C. Figure 3D presents the average fold change calculated for HM samples donated by individual women stored at 4 and −80 °C. Peak volumes of many DGs measured in samples stored at 4 °C were significantly higher than peak volumes of particular DGs measured in samples stored at −80 °C. However, the value of calculated fold change varied among women, as shown on the heat map presented in Figure 3D.

In samples stored at 4 °C, the highest decrease in the peak volume was detected for TGs with a maximum of two unsaturated bonds. According to this observation, the highest increase in concentration level of DGs with at most two unsaturated bonds was expected.

Interestingly, the highest increase in peak volume was observed for DGs with at least two unsaturated bonds. Linking the concentration level of individual TGs with their chemical structure and observed fold change clarified this discrepancy. The evaluation of TG structures suggests that DGs with at least two unsaturated bonds could be produced from the breakdown of TGs more frequently than others DG species. Moreover, HM contains a higher number of abundant TGs with more than two unsaturated bonds than those with less than two unsaturated chemical bonds (which are low-abundant TG species).^3^ Thus, even a small reduction of the content of several TGs with the same DG in the structure may produce a high increase in this particular DG species concentration.

The comparison of DG data obtained from samples stored at −20 and −80 °C demonstrated a similar trend to results obtained as an outcome of TG data analysis. The results showed that all of the detected MFs in samples stored at −20 and −80 °C had acceptable %RSD values (below 20%) and did not differ at a statistically significant level under a storage condition (except for DG34:3 (*p* < 0.005) and DG36:3 (*p* < 0.005) present in samples collected from W13A). The calculated %RSD values are presented in Table S2.

### Phospholipids

**Figure 4:**
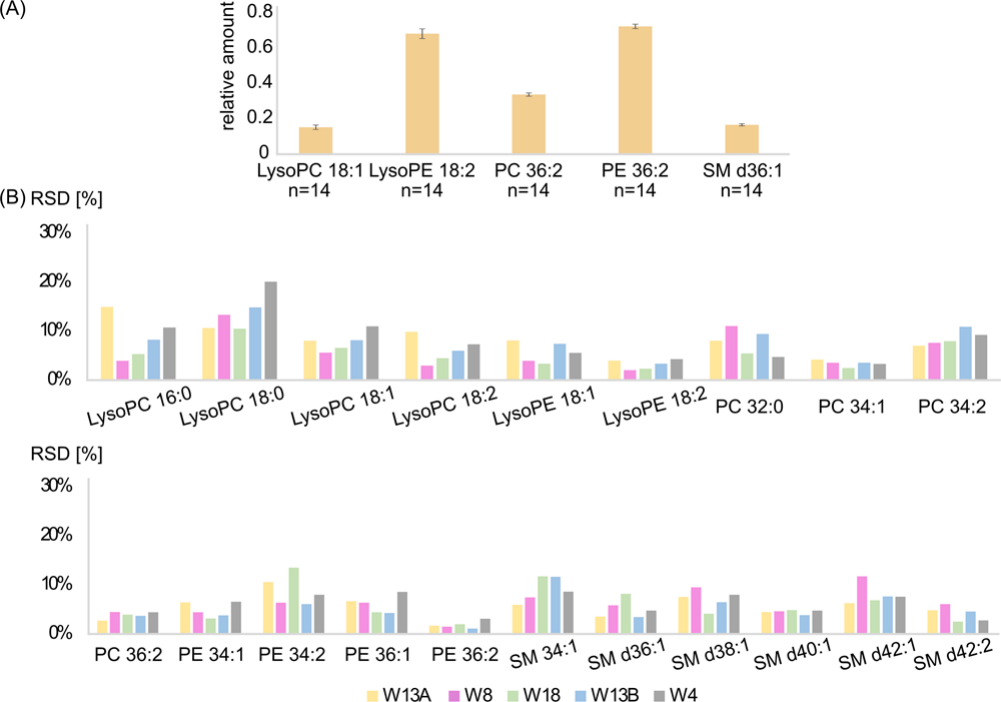
(A) Average relative amount of phospholipids detected in HM samples collected from W13A and stored under all studied conditions. Error bars represent standard deviations. (B) Calculated %RSD of phospholipids relative amount for samples stored at 4, −20, and −80 °C for all women (W13A, *n* = 14 (yellow bars); W8, *n* = 14 (pink bars); W18, *n* = 14 (green bars); W13B, *n* = 14 (blue bars); W4, *n* = 14 (gray bars)).

To further study the stability of HM lipidome, the stability of the phospholipids in the studied samples stored separately at 4, −20, and −80 °C during the different durations was investigated. Data examination included four lyso-glycerophosphocholines (lysoPCs), two lyso-glycerophosphocholines (lysoPEs), four diacylglycerophosphocholines (PCs), four diacylglycerophosphoethanoloamines (PEs), and six sphingomyelins (SMs). Here, we observed that the content of all examined phospholipids in the studied HM samples was not affected by any of the applied storage conditions. The relative amount of particular lipids was at the same level regardless of storage condition (Figure 4A).

Therefore, peak volumes of MFs detected in all samples stored under different temperatures and for various storage times (data from all storage conditions) were included to calculate %RSD values. The comparison of %RSD value of the percentage relative amount of lipid species within a specific lipid class revealed acceptable %RSD values (below 15%) among all samples stored at 4, −20, and −80 °C among all women (except lysoPC 18:0 with %RSD below 20% in samples donated by W4). A diagram with calculated %RSD values is presented in Figure 4B. The low value of calculated %RSD indicates no changes in phospholipid profiles regardless of condition storage. Thus, it might be assumed that the studied storage condition did not result in the alterations of HM phospholipid profiles.

### Inter-individual Differences between Women

Although a small number of samples do not allow for the reliable evaluation of the inter-individual differences between women, we used collected data to investigate if the observed lipidomic differences between women are maintained independently of the storage conditions. Here, PCA was used to visualize the variation in the lipid profiles among the collected HM samples stored at both −20 and −80 °C. The ANOVA unequal variance test was used to determine statistically significant changes (*p* < 0.05).

**Figure 5:**
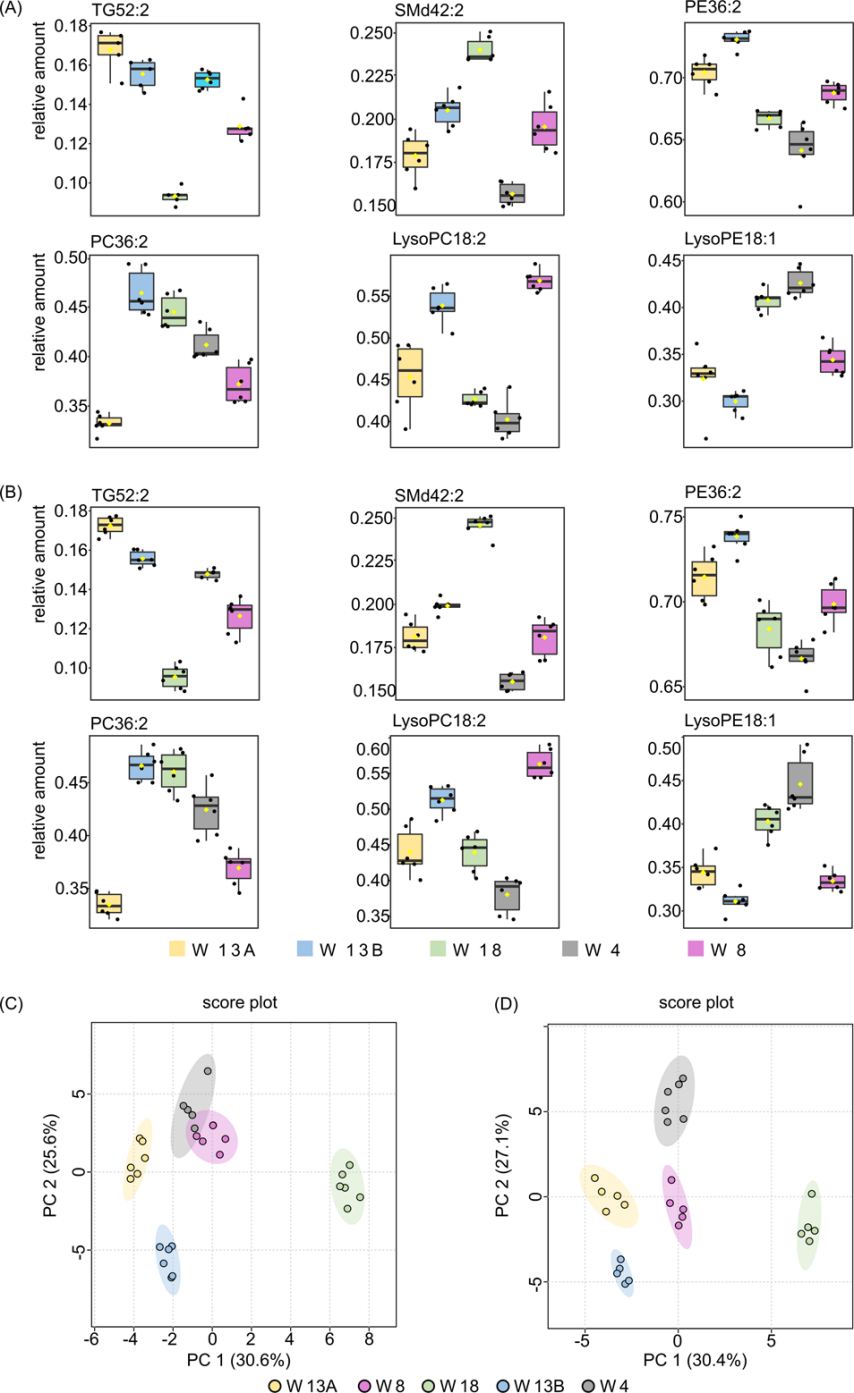
Comparison of the lipid relative amounts in samples collected from particular women and stored at (A) −80 °C and (B) −20 °C. The bar plots show the values of the relative amount (mean +/– standard deviation). The boxes range from the 25% and the 75% percentiles; the 5% and 95% percentiles are indicated as error bars; single data points are indicated by circles. Medians are indicated by horizontal lines within each box. PCA on the autoscaled relative amount of the TG and phospholipids of HM samples stored at (C) −20 °C and (D) −80 °C and collected from particular women: W13A (yellow circle); W8 (pink circles); W18 (green circles); W13B (blue circles); W4 (gray circles).

First, we analyzed the data set containing only TG profiles of HM samples. PCA showed a similar, clear grouping of milk samples of each participant indicating that inter-individual variance between different mothers was maintained, regardless of storage condition. The results of the unsupervised analysis are presented in the Supplementary Material section in Figure S1A,B. The results of the statistical analysis revealed that all the lipid components that were statistically different between samples collected from different women and stored at −80 °C were also indicated as statistically significant (*p* < 0.05) among samples stored at −20 °C. The differences in lipid composition between individual mothers were equal under both storage conditions: at −80 and −20 °C (Figure 5A,B). It should be mentioned that the *p* value obtained for a particular lipid depended on the storage condition, and one lipid that was not statistically significant in samples stored at −80 °C was indicated as statistically significantly changed in samples stored at −20 °C (TG 36:1, *p* < 0.005). A table with calculated *p* values is included in the Supporting Information (Table S3).

Next, we investigated the variation between HM phospholipids profiles. The results of the statistical analysis revealed the same statistically significantly different lipids between the studied samples stored under different conditions; however, the values of calculated *p* values were not equal (Table S4). The grouping of samples at PCA score plots slightly differed for −20 and −80 °C storage temperatures (Figure S2A,B). Points representing lipid profiles of HM samples collected by a woman in the 13th month of lactation (W13B) group separately from the samples of other participants in the PCA score plot were generated based on samples stored at −20 °C. Interestingly, in the PCA score plot generated based on samples stored at −80 °C, those samples group together with samples collected by a woman in the 8th and 13th month of lactation (W8 and W13A). Nevertheless, a detailed examination of the results revealed that the intra-individual differences can still be observed in samples stored in −20 °C and also in −80 °C (Figure 5A,B). The list of the phospholipid components that were statistically different among HM samples stored at −20 and −80 °C with calculated *p* values is shown in Tables S5.

We also investigated the grouping of lipid profiles containing both TG and phospholipid components. A PCA showed clear discrimination of samples collected from individual women in both score plots generated using samples stored at −20 and −80 °C. The obtained 2D PCA score plots are shown in Figure 5C,D, respectively. In Figure 5D, the points corresponding to samples from the early months of lactation (fourth and eighth, W4 and W8) tended to group in the middle part of the score plots, separately from HM samples from the 13th month of lactation (W13A and W13B) and 18th month lactation (W18), which were clustered in the left and right part of the score plots, respectively. When TG and GP lipid profiles are considered together (not individual lipid categories separately), discrimination of samples donated by an individual woman is clearer than considering them separately. Despite small overlap existing between samples collected by the women in the eighth (W8) and fourth month (W4) of lactation in the PCA score plot generated based on samples stored at −20 °C, the inter-individual variation was still visible. Due to the origin of samples labeled as 13A and 13B (both collected in the 13th month of lactation), overlapping of those samples in the score plots was expected. The separation of samples collected in the 13th month of lactation from two different women suggests that each woman has individual HM lipid composition and that the composition of milk depends on many factors, not only the time of lactation.

We observed that 4 days of storing samples at 4 °C significantly altered the concentration level of DGs and TGs (producing an increase and decrease in MS signal, respectively) when compared to storage at −80 °C. This is contrary to other researchers who studied the loss of TG in HM samples and stated that the TG concentration remains stable during storage at 4 °C. However, they investigated the level of the total concentration of TGs using a chemistry-routine analyzer with the colorimetric test in samples stored at 4C for only 48 h.^11^ In our study, the increased level of DGs with a simultaneously decreased level of TGs in samples stored at 4 °C indicates the hydrolysis of TGs and generation of DG lipid species as a consequence. The reduction of TG concentration levels and increased concentration levels of DGs may suggest lipase activity at 4 °C. The active lipolysis at −20 °C that results in a breakdown of the TG molecules, and consequently in the decrease in their content and increase in the content of DGs, was already observed in another study.^12^ In this study, we observed only slight (not statistically significant) differences in the concentration level of DGs and TGs contained in samples stored at −20 °C compared to samples stored at −80 °C. Those differences did not affect the results of lipidomic comparative analysis of HM samples collected by women being at different lactation periods. Comparison of lipid profiles revealed the same significant differences between individual mothers regardless of the storage temperature. However, it should be noted that the presented herein study included only samples that were stored at −20 °C for a maximum of 3 weeks and −80 °C for a maximum of 12 weeks. The research mentioned before, reporting that storage at −20 °C does not prevent lipid composition alterations, included storage of HM samples for several months.^10,12^ Reports suggesting that HM lipid composition is stable in samples stored at −20 °C for up to 3 months can be also found.^11,13^ Those discrepancies between scientific findings might be a result of different sample sizes, investigated features (total fat, total TGs, or fatty acid content), and applied analytical techniques (human milk analyzer, gas chromatography, thin-layer chromatography, and colorimetric test). Further studies on lipidome stability at −20 °C are necessary to clarify lipid composition alteration under this condition. Fat loss might be attributed to its adherence to the container, lipolysis, or lipid peroxidation.^22^ Thus, all those features should be included and investigated on a representative number of HM samples.

The observed alterations were not at the same levels in studied women. The content of TGs that underwent breakdown was different in the samples collected from different women. These results indicate that alteration of lipid composition during storage can be associated with lipase activity present in HM. The huge inter-individual variability among HM lipase activity was already mentioned in a previous study.^23^ Generally, the activity of the lipase depends on various factors, e.g., the presence of compounds that decrease HM lipase activity (e.g., endogenous proteases) or milk fat globule (MFG) size distribution and composition.^24,25^ Therefore, observed differences in the TG disruption might be a result of all these factors that have an impact on lipase activity. Additional studies focused on the inter-individual variation of lipase activity may be helpful to clarify those differences.

### Freeze–Thawing Cycles

Multiple freeze–thawing of the biological sample can result in the degradation of lipid components and falsify the data as a consequence. Nevertheless, the study of the impact of such sample treatment on the stability of HM lipidome samples by the analysis of each lipid species separately was not carried out so far. We have examined the effect of up to three freeze–thaw cycles on HM lipidome stability. Three samples collected from each woman underwent freeze–thaw cycles over 1 day, and subsequently all 15 samples were analyzed in a single batch. We compared the TG, DG, and phospholipid profiles of HM samples separately to investigate the impact of freeze–thawing cycles on HM lipid stability. The PCA was implemented to investigate variation in HM lipidomic profiles.

**Figure 6:**
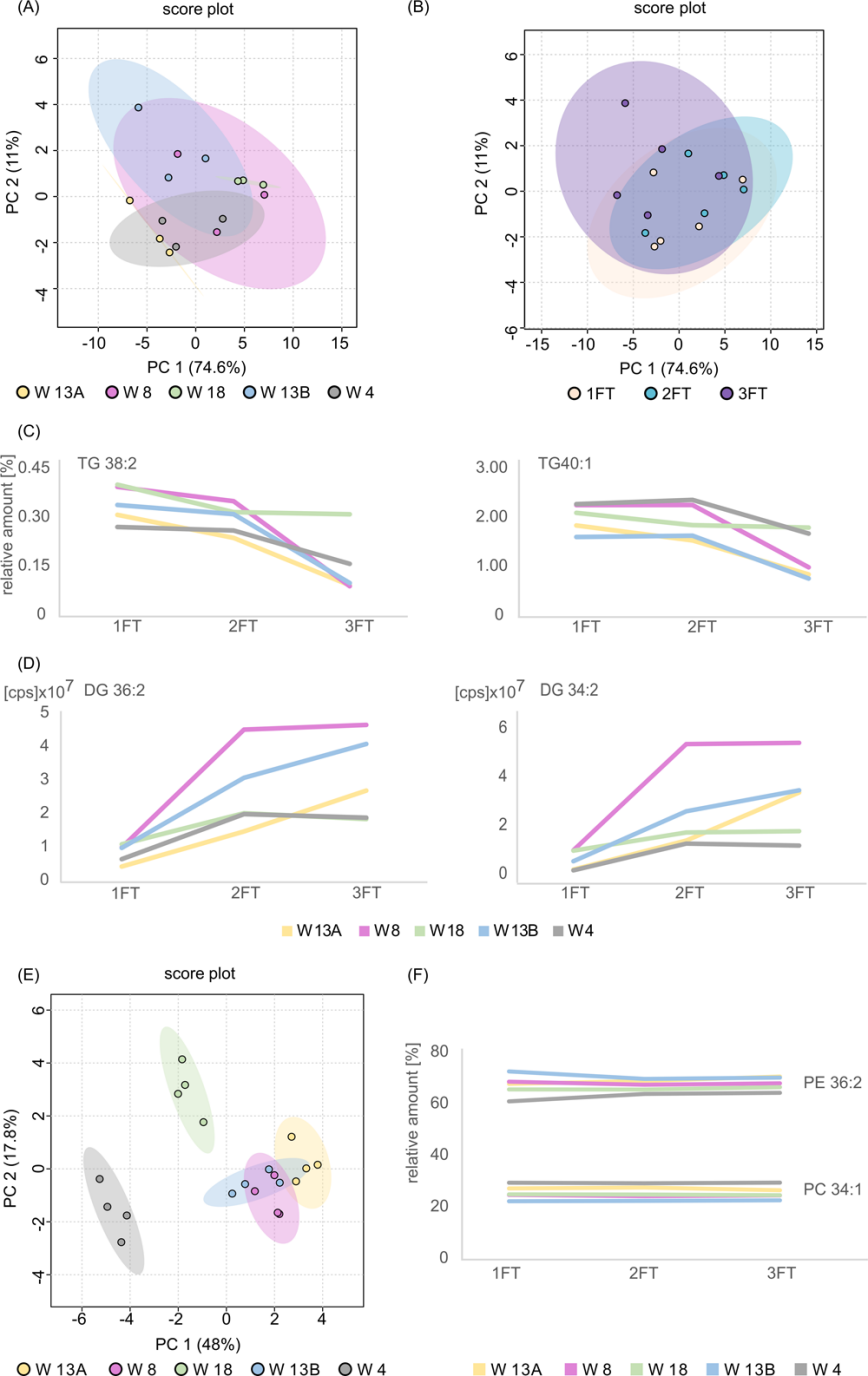
PCA on the autoscaled and logarithmic peak volume of the TGs detected in HM samples that underwent up to three freeze–thaw cycles (FT). (A) Samples are colored according to their origin: W13A (yellow circle), W8 (pink circles), W18 (green circles), W13B (blue circles), and W4 (gray circles) or (B) according to the number of freeze–thaw cycles. (C) Variation in the relative amount of TG species detected in HM samples that underwent up to three freeze–thaw cycles. (D) Variation in the peak volumes of DG species detected in HM samples that underwent up to three freeze–thaw cycles. (E) PCA on the autoscaled relative amount of the phospholipid species detected in HM samples that underwent up to three freeze–thaw cycles. Samples are colored according to the donor: W13A (yellow circle), W8 (pink circles), W18 (green circles), W13B (blue circles), and W4 (gray circles). (F) Variation in the relative amount of phospholipid species detected in HM samples that underwent up to three freeze–thaw cycles.

First, raw data sets containing information on TG peak volumes detected in all studied HM samples that underwent freeze–thaw cycles were analyzed. PCA was applied to visualize the variance between the analyzed samples. The obtained score plots are presented in Figure 6A,B. The discrimination of samples that underwent three freeze–thaw cycles is visible. However, a clear distinction of samples freezing and thawing three times was obtained only for samples that were donated by women marked as W8, W13A, and W13B. Clustering of the points corresponding to samples collected from women W4 and W18 is showing that at least at the level detectable by PCA, three freeze–thaw cycles have not produced an important alteration in TG composition. Further, investigation of the data set revealed that the relative amount of some detected MF volumes corresponding to TGs has decreased, whereas MF volumes corresponding to DGs have increased with subsequent freeze–thaw cycles. The table with the relative amount of particular TGs detected in freeze–thawed samples is included in the Supporting Information in Table S5, whereas the changes in the contribution of lipid species among class are shown in Figure 6C. The trend of increasing peak volume of DGs with subsequent freeze–thaw cycles is presented in Figure 6D (detailed information about peak volume of DGs with subsequent freeze–thaw cycles is included in the Supporting Information in Table S6).

Freeze–thaw cycles resulted in the alteration of the lipid concentration level in HM samples. The decrease in TG concentration levels indicates the progressive breakdown of those molecules to DGs with subsequent freeze–thaw cycles. The increasing concentration level of DGs with subsequent freeze–thaw cycles confirmed the validity of this theory. According to the literature, freeze–thaw cycles do not activate or inactivate HM lipases but disrupt the milk fat globule membrane and simplify the access of HM lipases to the TGs contained within the core of the fat globule that could be the reason for their progressive hydrolysis.^23^

PCA was performed also to investigate the differences and similarities between phospholipid profiles of HM samples that underwent freeze–thawing cycles. The obtained 2D PCA score plot is shown in Figure 6E. Points representing the same subject tended to cluster, irrespectively to the number of freeze–thaw cycles, on the plane of the two first principal components (65.8% of the total explained variance). Clustering of the points corresponding to samples collected from the same woman is showing that at least at the level detectable by PCA, three freeze–thawing cycles did not have a significant effect on the phospholipid profiles of HM samples. Further analysis, including calculation of %RSD of the MF volumes detected in samples that underwent a different number of freeze–thaw cycles, revealed acceptable data variation (%RSD values below 20%) among each woman. A table with calculated %RSD values is included in the Supporting Information in Table S7. The stability of the content level of two phospholipids regardless of the number of freeze–thawing cycles is demonstrated in Figure 6F. While significant changes in phospholipid profiles were not observed, it can be assumed that up to three freeze–thawing cycles do not affect the output of the HM phospholipid analysis.

## Conclusions

The results shown here indicate that storing HM samples for up to 12 weeks at −80 °C might be considered to be suitable to ensure HM lipidome stability, while for a shorter storage period (<3 weeks), storage at −20 °C can be considered. We have shown that there are no statistically significant differences between the lipid profiles of HM samples stored at −20 °C (for up to 3 weeks) and −80 °C (for up to 12 weeks), and the inter-individual variance regardless of sample storage condition was maintained. However, both storage at 4 °C for up to 4 days and subsequent freeze–thaw cycles (up to three) affected the sample lipid composition and caused the decrease in TG peak volumes. The short-term storage at 4 °C still seems to provide samples usable for phospholipid analysis since significant changes in phospholipid profiles were not observed regardless of sample storage conditions and also number of freeze–thawing cycles. It can be assumed that the storage of HM samples under studied storage conditions (4 °C for up to 4 days, −20 °C for up to 3 weeks, and −80 °C for up to 3 months) and up to three freeze–thawing cycles does not affect the output of the HM phospholipid analysis. Due to the particular components of the sample being stable under different conditions, storage conditions should be selected according to lipids of interest. We strongly recommend storing HM samples at temperatures of at least −20 °C for short and −80 °C for longer periods and avoiding the number of freeze and thaw cycles to reduce potential biological misinterpretation of HM lipidomics data.

## Supporting Information Available

Details of reagents and materials, sample preparation LC–MS analysis, and data treatment; (Table S1) %RSD values calculated for TG relative amounts among all samples stored at −20 and −80 °C; (Table S2) %RSD values calculated for DG peak volumes detected among all samples stored at −20 and −80 °C; (Figure S1) PCA of the TG profiles of HM samples stored at −20 and −80 °C and collected from individual women; (Table S3) TGs statistically significantly different between the individual mothers according to the Mann–Whitney test (unpaired) (*p* < 0.05); (Table S4) phospholipids statistically significantly different between the individual mothers according to the Mann–Whitney test unpaired (*p* < 0.05); (Figure S2) PCA of the phospholipids profiles of HM samples stored at −20 and −80 °C and collected from particular women; (Table S5) relative amounts of TGs detected in samples that underwent up to three freeze–thaw cycles; (Table S6) peak volumes of DGs detected in samples that underwent up to three freeze–thaw cycles; and (Table S7) %RSD values calculated for phospholipid relative amounts in samples that underwent three freeze–thawed cycles (PDF)
