# Supplementary material for: The Influence of Storage on Human Milk Lipidome Stability for Lipidomic Studies
Source: J Proteome Res. 2021 Dec 29;21(2):438–46. doi: 10.1021/acs.jproteome.1c00760 (PMC8822481; doi:10.1021/acs.jproteome.1c00760)
Supplement: Supplementary file 1 — pr1c00760_si_001.pdf [file pr1c00760_si_001.pdf]

The influence of storage on the human milk lipidome stability for the lipidomic studies – Supplementary materials

Dorota Garwolińska[1], Michał Młynarczyk[1], Agata Kot-Wasik[1], and Weronika Hewelt-Belka[1,*]

[1]Department of Analytical Chemistry, Faculty of Chemistry, Gdańsk University of Technology, Gabriela Narutowicza 11/12, 80-233 Gdańsk, Poland

Corresponding Author
*e-mail: werbelka@pg.edu.pl.

Table of contents

	Supporting Information	Page
	Reagents and materials.	S-2
	Sample preparation	S-2
	LC-MS analysis.	S-2
	Data treatment.	S-2
Table S1	The %RSD values calculated for TG relative amounts among all samples stored at -20°C and -80°C	S-3
Table S2	The %RSD values calculated for DG peak volumes detected among all samples stored at -20°C and -80°C	S-4
Figure S1	The PCA of the TG profiles (%relative amount) of HM samples stored at (A) -20°C and (B) -80°C and collected from individual women: W13A (yellow circle); W8 (pink circles); W18 (green circles); W13B (blue circles); W4 (grey circles).	S-4
Table S3	TGs statistically significantly different between the individual mothers accordingly to the Mann-Whitney test unpaired (p<0.05).	S-5
Table S4	Phospholipids statistically significantly different between the individual mothers accordingly to the Mann-Whitney test unpaired (p<0.05).	S-6
Figure S2	PCA of the phospholipids profiles of HM samples stored at (A) -20°C and (B) -80°C and collected from particular women: W13A (yellow circle); W8 (pink circles); W18 (green circles); W13B (blue circles); W4 (grey circles).	S-6
Table S5	The relative amounts of TGs detected in samples that underwent up to three freeze-thawed cycles (1FT – one freeze-thaw cycle, 2FT – two freeze-thaw cycles, 3 – three freeze-thaw cycles)	S-7
Table S6	The peak volumes of DGs detected in samples that underwent up to three freeze-thawed cycles (1FT – one freeze-thaw cycle, 2FT – two freeze-thaw cycles, 3 – three freeze-thaw cycles)	S-8
Table S7	The %RSD values calculated for phospholipid relative amounts in samples that underwent three freeze-thawed cycles	S-9

Reagents and materials. LC−MS-grade methanol, HPLC-grade chloroform and n-hexane were purchased from Merck (Darmstadt, Germany). HPLC-grade 2-propanol, ammonium formate (99.9% purity), formic acid and ammonia p.a. were purchased from Sigma-Aldrich (St. Louis, MO, USA). Deionized water was purified on an HLP5 system (Hydrolab, Wislina, Poland).

Sample preparation. After 10s of vigorous vortexing, 100 μL HM sample was transferred and mixed with 900 μL 1% formic acid in methanol in a polypropylene tube. Next, sample was vortex for 30 s, and centrifuge for 10 min at 10, 732×g. The 900 μL of obtained supernatant was transferred to a Hybrid-SPE-Phospholipid (bed weight 20 mg) cartridge (Supelco, Sigma Aldrich, St. Louis, MO, USA). After supernatant loading, the stationary phase was washed with the use of methanol and 2-propanol, and next phospholipids were eluted with 2 mL 5% ammonia in methanol. The obtained phospholipid extract was evaporated to dryness under a nitrogen stream and dissolved in the 50-fold diluted extract obtained in the LLE-based step. LLE-based step was performed based on modified Bligh and Dyer extraction. After 10s of vigorous vortexing, 225 μL HM sample was transferred and mixed with 950 μL chloro-form/methanol (1/2, v/v) in a borosilicate glass tube with a PTFE cap. After 10 s of vortexing, 310 μL chloroform and 310 μL water were added. To separate the organic phases containing lipids, the sample was centrifuged for 10 min at 5000×g. The 20 μL lower organic fraction was transferred to glass test tube and mix with 980 μL methanol containing IS (1μg/mL, PC 18:0/18:0)). The 100 μL prepared diluted lipid extract was used to dissolve phospholipid extract evaporated to dryness in previous SPE-based step. The obtained enriched-diluted extract was transferred to a chromatographic vial and analyzed by RP-LC-Q-TOF-MS.

LC-MS analysis. Briefly, lipids were chromatography separated on a on a reversed-phase column (Poroshell 120 EC-C8, 2.1×150 mm, 1.9 μm particle size, Agilent) with a 0.2 μm in-line filter. The column was maintained at 45°C. The mobile phase consisted of a component A: a mixture of 5mM ammonium formate in water and methanol (1/4, v/v) and component B: a mixture of 5mM ammonium formate in water, n-hexane and 2-propanol (1/20/79, v/v/v) and was pumped with a flow rate 0.5 mL/min using gradient program. Total run time was 30.5 min, and the injection volume was 0.5 μL.

The MS data were collected in positive ion mode using the SCAN acquisition mode in a range from 200 to 1700 m/z in high-resolution mode (4 GHz). The remaining MS parameters were set as follows: capillary voltage of 3500 V and fragmentor voltage of 120 V, nebulizer gas pressure of 35 psi, drying gas flow rate of 10 L/min and temperature of 300°C.

Data treatment. The Batch Targeted Feature Extraction algorithm was performed with the following parameters: positive ions, charge carriers—H+, Na+, NH4 +; match tolerance, 15 ppm; retention time, 0.3 min; Gaussian smoothing before extracted ion chromatogram extraction (EIC) filtering on peak height, 1000 counts. The .cef files were ex-ported and imported to Mass Profiler Professional 15.1 software (Agilent Technologies, Santa Clara, CA, USA) for data alignment and filtration. Missing values were exported as missing. The alignment parameters were set as follows: alignment slope = 0.0%; alignment intercept = 0.4 min; mass tolerance slope = 20.0 ppm; intercept = 2.0 mDa. Filtration was based on the peak characteristics (peak symmetry, tailing), frequency (the MFs remained in the dataset if they were present in 100% of the samples in at least one specified group (specific storage condition)) and the QC %RSD (MFs remained if %RSD <30% in all the QC samples). The MFs that were present in the extraction blank, with the average peak volume higher than 10% of the average peak volume in the real samples, were removed.

Table S2. The %RSD values calculated for TG relative amounts among all samples stored at -20°C and -80°C

Compound	storing at-20°C					storing at -80°C				
	W13A	W8	W18	W13B	W4	W13A	W8	W18	W13B	W4
TG30:0	26%	13%	13%	24%	16%	9%	17%	20%	11%	13%
TG34:1	12%	9%	7%	10%	13%	12%	13%	17%	11%	14%
TG36:0	22%	15%	14%	16%	20%	8%	10%	14%	12%	9%
TG36:1	13%	5%	10%	12%	12%	11%	11%	15%	12%	11%
TG38:0	10%	5%	7%	17%	13%	10%	8%	12%	11%	9%
TG38:1	11%	5%	11%	10%	13%	9%	11%	11%	9%	10%
TG38:2	5%	10%	7%	2%	15%	13%	12%	18%	14%	13%
TG40:0	9%	15%	9%	10%	10%	18%	12%	15%	8%	11%
TG40:1	7%	8%	5%	8%	12%	5%	6%	8%	8%	7%
TG42:1	9%	9%	4%	9%	13%	3%	5%	4%	7%	6%
TG44:1	4%	4%	3%	1%	1%	8%	5%	4%	5%	5%
TG44:2	8%	9%	3%	7%	10%	4%	3%	3%	6%	4%
TG46:1	8%	8%	8%	8%	2%	7%	6%	5%	4%	5%
TG46:2	4%	3%	6%	5%	4%	7%	3%	4%	7%	6%
TG46:3	6%	7%	2%	8%	8%	3%	6%	4%	4%	4%
TG48:2	5%	4%	9%	4%	3%	4%	3%	6%	5%	4%
TG48:3	4%	5%	2%	4%	2%	6%	7%	1%	5%	5%
TG48:4	3%	7%	4%	5%	5%	6%	4%	6%	3%	5%
TG50:2	5%	5%	7%	5%	6%	9%	6%	4%	6%	8%
TG50:3	6%	2%	4%	6%	4%	8%	7%	6%	3%	6%
TG50:4	2%	5%	3%	4%	7%	8%	10%	2%	2%	4%
TG52:2	3%	6%	5%	2%	2%	6%	6%	5%	5%	3%
TG52:3	4%	2%	4%	2%	4%	3%	3%	9%	2%	6%
TG52:4	7%	6%	4%	7%	6%	8%	6%	1%	4%	4%
TG54:2	14%	15%	20%	7%	11%	18%	12%	15%	8%	7%
TG54:3	8%	9%	9%	9%	7%	10%	11%	6%	5%	6%
TG54:4	7%	7%	5%	7%	7%	9%	3%	4%	3%	7%

Table S2. The %RSD values calculated for DG peak volumes detected among all samples stored at -20°C and -80°C

	storing at-20°C					storing at -80°C				
Compound	W13A	W8	W18	W13B	W4	W13A	W8	W18	W13B	W4
DG24:0	14%	13%	11%	13%	10%	20%	6%	9%	13%	14%
DG 26:0	13%	8%	8%	6%	10%	12%	8%	7%	8%	12%
DG 28:0	6%	4%	4%	5%	9%	11%	7%	9%	8%	11%
DG 28:1	13%	7%	9%	15%	14%	15%	13%	21%	15%	10%
DG30:0	13%	8%	9%	7%	9%	15%	6%	4%	11%	16%
DG30:1	3%	6%	3%	3%	12%	4%	9%	9%	8%	6%
DG30:2	8%	7%	3%	10%	19%	7%	14%	10%	13%	6%
DG32:1	5%	4%	5%	7%	8%	5%	5%	2%	3%	4%
DG32:2	2%	7%	4%	4%	19%	12%	8%	5%	7%	10%
DG34:1	1%	3%	5%	4%	8%	3%	3%	4%	3%	4%
DG34:2	4%	2%	4%	3%	7%	3%	4%	3%	2%	3%
DG34:3	4%	13%	18%	10%	21%	7%	7%	5%	5%	4%
DG36:2	12%	8%	9%	7%	17%	17%	10%	15%	14%	16%
DG36:3	7%	3%	8%	5%	14%	4%	6%	3%	8%	6%
DG36:4	13%	5%	6%	10%	15%	17%	4%	4%	4%	11%

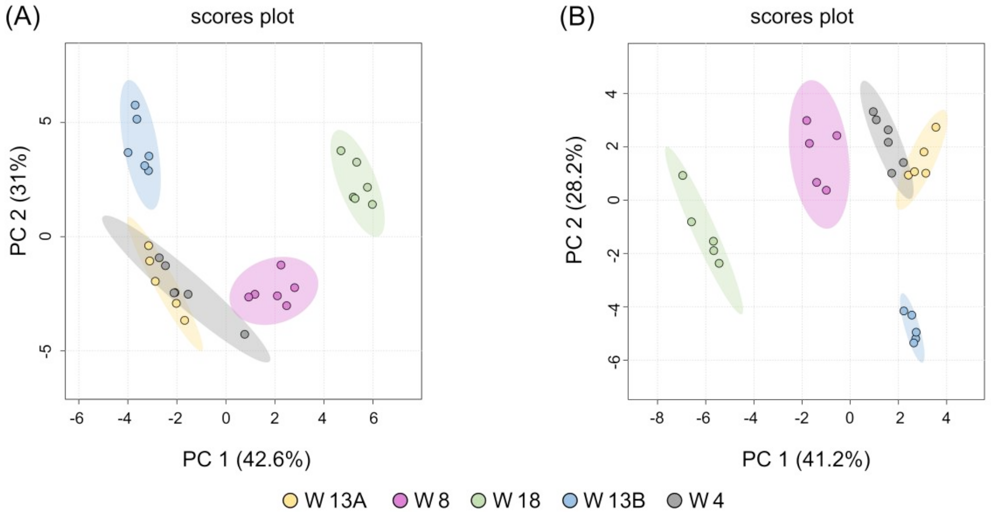

Figure S1. The PCA of the TG profiles (%relative amount) of HM samples stored at (A) -20°C and (B) -80°C and collected from individual women: W13A (yellow circle); W8 (pink circles); W18 (green circles); W13B (blue circles); W4 (grey circles).

Table S3. TGs statistically significantly different between the individual mothers accordingly to the Mann-Whitney test unpaired ($p<0.05$).

	p-value	
compound	storing at-20°C	storing at -80°C
TG30:0	2.71E-04	7.31E-04
TG34:1	1.64E-03	7.07E-03
TG36:0	1.39E-03	5.55E-04
TG36:1	4.92E-03	not significant
TG38:0	2.19E-04	6.85E-04
TG38:1	2.62E-03	9.51E-03
TG38:2	9.65E-04	6.80E-03
TG40:0	1.58E-04	1.57E-03
TG40:1	2.11E-04	7.56E-04
TG42:1	1.84E-04	4.44E-04
TG44:1	6.90E-05	1.24E-03
TG44:2	6.24E-05	1.46E-04
TG46:1	1.75E-04	1.30E-04
TG46:2	5.88E-05	1.85E-04
TG46:3	7.87E-05	1.67E-04
TG48:2	4.49E-04	3.36E-04
TG48:3	3.41E-05	1.37E-04
TG48:4	3.50E-05	1.20E-04
TG50:2	2.07E-04	2.00E-03
TG50:3	1.13E-04	3.19E-04
TG50:4	2.25E-05	9.16E-05
TG52:2	2.23E-05	3.05E-04
TG52:3	1.00E-04	2.14E-04
TG52:4	1.55E-04	2.07E-04
TG54:2	2.25E-04	5.49E-04
TG54:3	6.16E-05	1.78E-04
TG54:4	4.28E-05	2.11E-04
TG54:5	7.61E-05	2.14E-04

Table S4. Phospholipids statistically significantly different between the individual mothers accordingly to the Mann-Whitney test unpaired (p<0.05).

	p-value	
compound	storing at-20°C	storing at -80°C
LysoPC16:0	0.000257	0.000162
Lyso-PC18:0	0.000741	0.000216
Lyso-PC18:1	7.09E-05	0.000113
Lyso-PC18:2	3.48E-05	7.56E-05
Lyso-PE18:1	4.69E-05	5.38E-05
LYsoPE18:2	4.69E-05	5.38E-05
PC32:0	5.85E-05	0.02481
PC34:1	5.89E-05	3.11E-05
PC34:2	3.13E-05	5.1E-05
PC36:2	5.51E-05	2.89E-05
PE34:1	2.53E-05	2.95E-05
PE34:2	0.000106	0.000102
PE36:1	0.000176	0.000154
PE36:2	8.29E-05	2.2E-05
SMd34:1	0.008044	0.006134
SMd36:1	1.99E-05	0.000455
SMd38:1	0.019059	0.003107
SMd40:1	0.001714	0.020512
SMd42:1	0.000802	0.015676
SMd42:2	3.1E-05	4.84E-05

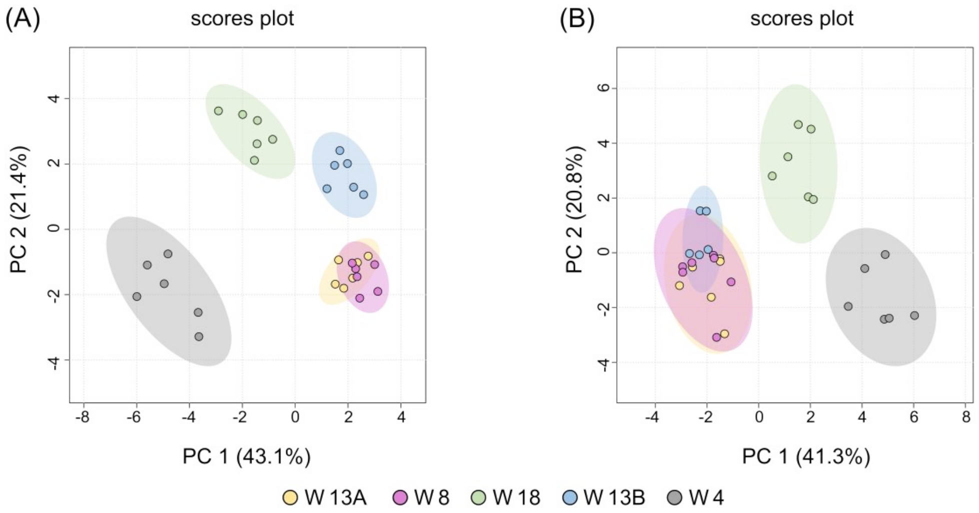

Figure S2. PCA of the phospholipids profiles of HM samples stored at (A) -20°C and (B) -80°C and collected from particular women: W13A (yellow circle); W8 (pink circles); W18 (green circles); W13B (blue circles); W4 (grey circles).

Table S5. The relative amounts of TGs detected in samples that underwent up to three freeze-thawed cycles (1FT – one freeze-thaw cycle, 2FT – two freeze-thaw cycles, 3 – three freeze-thaw cycles)

	W13A			W8			W18			W1B			W4		
Compound	1FT	2FT	3FT	1FT	2FT	3FT	1FT	2FT	3FT	1FT	2FT	3FT	1FT	2FT	3FT
TG34:1	0.11%	0.08%	0.01%	0.19%	0.14%	0.00%	0.14%	0.11%	0.11%	0.12%	0.10%	0.00%	0.14%	0.13%	0.07%
TG36:0	0.76%	0.65%	0.42%	0.79%	0.73%	0.37%	1.06%	0.82%	0.87%	0.48%	0.44%	0.26%	0.90%	0.89%	0.64%
TG36:1	0.40%	0.30%	0.12%	0.49%	0.44%	0.10%	0.42%	0.33%	0.32%	0.39%	0.34%	0.10%	0.41%	0.38%	0.22%
TG38:0	1.11%	1.03%	0.93%	1.12%	1.41%	0.83%	1.70%	1.63%	1.63%	0.76%	0.85%	0.53%	1.36%	1.47%	0.97%
TG38:1	1.35%	1.05%	0.47%	1.45%	1.26%	0.39%	1.06%	0.89%	0.86%	1.19%	1.11%	0.41%	1.25%	1.21%	0.77%
TG38:2	0.31%	0.24%	0.10%	0.40%	0.35%	0.09%	0.41%	0.32%	0.31%	0.34%	0.31%	0.10%	0.28%	0.26%	0.16%
TG40:0	1.80%	1.75%	1.62%	1.83%	2.03%	1.51%	2.38%	2.21%	2.20%	1.07%	1.18%	0.87%	1.71%	1.93%	1.57%
TG40:1	1.87%	1.56%	0.87%	2.28%	2.28%	1.01%	2.12%	1.87%	1.82%	1.63%	1.66%	0.79%	2.30%	2.39%	1.70%
TG42:1	3.37%	3.05%	2.16%	4.62%	4.58%	2.92%	4.21%	4.07%	4.02%	2.80%	3.00%	1.80%	4.46%	4.80%	3.70%
TG44:1	6.38%	6.70%	5.92%	6.65%	6.40%	6.10%	5.74%	5.87%	5.79%	4.98%	5.38%	4.39%	6.33%	6.45%	6.06%
TG44:2	2.14%	2.10%	1.72%	2.67%	3.03%	2.03%	3.34%	3.10%	3.14%	1.86%	2.03%	1.42%	2.08%	2.36%	1.84%
TG46:1	8.57%	8.56%	8.85%	7.71%	7.17%	8.33%	6.21%	6.55%	6.60%	5.94%	6.13%	5.96%	7.87%	7.59%	8.15%
TG46:2	3.47%	3.71%	3.34%	4.57%	4.80%	4.22%	4.67%	4.66%	4.62%	3.23%	3.69%	3.05%	3.90%	4.34%	3.77%
TG46:3	1.09%	1.09%	0.94%	1.43%	1.65%	1.21%	1.81%	1.68%	1.69%	1.28%	1.31%	1.11%	1.13%	1.28%	1.06%
TG48:2	5.98%	6.06%	6.01%	6.68%	6.54%	7.12%	6.07%	6.38%	6.50%	5.67%	5.90%	5.64%	7.08%	7.18%	7.35%
TG48:3	2.29%	2.39%	2.21%	3.79%	4.19%	3.55%	4.69%	4.65%	4.69%	2.83%	3.30%	2.76%	3.01%	3.49%	2.95%
TG48:4	0.68%	0.68%	0.60%	1.16%	1.31%	1.03%	1.79%	1.69%	1.69%	1.12%	1.07%	0.88%	0.73%	0.82%	0.70%
TG50:2	8.78%	8.80%	9.52%	7.28%	6.75%	8.23%	6.03%	6.44%	6.48%	7.35%	6.87%	8.10%	7.42%	6.91%	7.89%
TG50:3	3.68%	3.83%	3.80%	3.98%	4.38%	4.38%	4.45%	4.67%	4.69%	3.70%	4.02%	3.93%	2.98%	3.38%	3.17%
TG50:4	1.05%	1.09%	1.05%	1.31%	1.56%	1.33%	1.94%	1.89%	1.94%	1.49%	1.41%	1.31%	0.84%	0.92%	0.85%
TG52:2	16.72%	16.68%	18.89%	12.74%	10.96%	14.73%	10.41%	9.82%	9.91%	15.08%	#####	15.96%	14.34%	11.94%	15.74%
TG52:3	11.37%	11.09%	12.19%	8.68%	8.52%	10.05%	7.97%	8.67%	8.08%	10.53%	9.27%	11.30%	9.38%	8.61%	9.74%
TG52:4	3.82%	3.92%	3.91%	3.90%	4.19%	3.98%	4.22%	4.31%	4.40%	4.52%	4.53%	4.65%	2.53%	2.79%	2.32%
TG54:2	3.74%	3.87%	4.24%	3.17%	3.76%	3.82%	3.04%	3.02%	3.10%	5.23%	5.71%	5.94%	4.05%	4.63%	4.26%
TG54:3	4.42%	4.73%	4.95%	5.38%	5.59%	6.22%	5.79%	5.71%	5.87%	7.57%	7.85%	8.76%	7.31%	7.60%	7.85%
TG54:4	2.96%	3.21%	3.34%	3.69%	3.72%	4.18%	4.98%	5.13%	5.18%	5.37%	5.52%	6.14%	4.32%	4.35%	4.47%
TG54:5	1.75%	1.79%	1.85%	2.04%	2.26%	2.26%	3.35%	3.49%	3.49%	3.48%	3.64%	3.85%	1.90%	1.88%	2.05%

Table S6. The peak volumes of DGs detected in samples that underwent up to three freeze-thawed cycles (1FT – one freeze-thaw cycle, 2FT – two freeze-thaw cycles, 3 – three freeze-thaw cycles)

	W13A			W8			W18			W1B			W4		
Compound	1FT	2FT	3FT	1FT	2FT	3FT	1FT	2FT	3FT	1FT	2FT	3FT	1FT	2FT	3FT
DG24:0	9.25E+05	1.86E+06	3.52E+06	3.44E+06	1.17E+07	8.35E+06	2.94E+06	3.59E+06	4.06E+06	1.21E+06	3.28E+06	3.77E+06	1.45E+06	3.86E+06	3.26E+06
DG 26:0	1.78E+06	4.52E+06	8.88E+06	5.63E+06	1.96E+07	1.60E+07	4.97E+06	5.98E+06	7.03E+06	2.42E+06	7.08E+06	7.75E+06	2.19E+06	6.28E+06	6.08E+06
DG 28:0	2.19E+06	5.51E+06	1.20E+07	6.97E+06	2.50E+07	2.32E+07	5.92E+06	6.26E+06	8.74E+06	3.23E+06	9.00E+06	1.05E+07	2.57E+06	7.57E+06	7.62E+06
DG 28:1	9.44E+05	3.28E+06	5.68E+06	3.28E+06	1.43E+07	1.05E+07	2.39E+06	3.58E+06	4.12E+06	1.66E+06	5.85E+06	5.98E+06	1.44E+06	5.06E+06	4.60E+06
DG 28:2	2.80E+05	9.94E+05	1.70E+06	1.03E+06	4.92E+06	3.39E+06	1.13E+06	1.57E+06	1.86E+06	5.38E+05	2.05E+06	2.11E+06	4.26E+05	1.44E+06	1.26E+06
DG30:0	2.05E+06	4.77E+06	1.15E+07	4.99E+06	1.75E+07	1.73E+07	4.46E+06	5.29E+06	5.95E+06	2.71E+06	7.60E+06	9.44E+06	2.01E+06	4.63E+06	5.12E+06
DG30:1	2.44E+06	9.12E+06	1.66E+07	1.07E+07	4.60E+07	4.00E+07	7.33E+06	1.12E+07	1.49E+07	4.42E+06	1.76E+07	1.89E+07	4.12E+06	1.49E+07	1.48E+07
DG30:2	7.57E+05	3.18E+06	5.92E+06	3.93E+06	1.92E+07	1.55E+07	3.61E+06	6.09E+06	7.28E+06	1.57E+06	6.56E+06	7.17E+06	1.17E+06	4.89E+06	4.82E+06
DG32:1	4.50E+06	1.38E+07	2.79E+07	1.30E+07	5.38E+07	5.07E+07	1.16E+07	1.66E+07	1.85E+07	7.61E+06	2.44E+07	3.01E+07	4.82E+06	1.49E+07	1.53E+07
DG32:2	1.48E+06	5.22E+06	1.11E+07	5.16E+06	2.44E+07	2.16E+07	5.98E+06	9.42E+06	1.05E+07	2.74E+06	1.07E+07	1.37E+07	1.67E+06	5.51E+06	5.69E+06
DG34:1	1.52E+07	4.41E+07	8.65E+07	2.96E+07	1.23E+08	1.21E+08	2.25E+07	3.31E+07	3.37E+07	2.25E+07	6.57E+07	9.06E+07	1.52E+07	4.39E+07	4.11E+07
DG34:2	5.82E+06	1.78E+07	3.75E+07	1.37E+07	5.74E+07	5.79E+07	1.36E+07	2.11E+07	2.16E+07	9.24E+06	2.98E+07	3.84E+07	5.49E+06	1.65E+07	1.57E+07
DG34:3	1.08E+06	3.91E+06	8.51E+06	3.26E+06	1.48E+07	1.44E+07	3.19E+06	5.49E+06	5.48E+06	2.33E+06	8.36E+06	1.12E+07	8.98E+05	3.17E+06	3.09E+06
DG36:2	4.69E+06	1.52E+07	2.74E+07	1.04E+07	4.56E+07	4.70E+07	1.13E+07	2.06E+07	1.89E+07	1.03E+07	3.12E+07	4.13E+07	6.90E+06	2.03E+07	1.93E+07
DG36:3	3.08E+06	9.54E+06	1.87E+07	7.61E+06	3.61E+07	3.59E+07	1.03E+07	1.78E+07	1.79E+07	6.70E+06	2.26E+07	3.01E+07	3.56E+06	1.22E+07	1.16E+07
DG36:4	9.38E+05	3.84E+06	7.25E+06	3.01E+06	1.63E+07	1.48E+07	4.49E+06	8.49E+06	8.12E+06	2.96E+06	1.06E+07	1.34E+07	1.01E+06	3.62E+06	3.52E+06

Table S7. The %RSD values calculated for phospholipid relative amounts in samples that underwent three freeze-thawed cycles

	W13A	W8	W18	W1B	W4
LysoPC16:0	18%	3%	9%	9%	5%
Lyso-PC18:1	4%	5%	5%	6%	3%
Lyso-PC18:2	11%	3%	6%	3%	4%
Lyso-PE18:1	12%	11%	7%	3%	18%
LYsoPE18:2	5%	6%	5%	12%	14%
PC32:0	11%	10%	7%	13%	4%
PC34:1	5%	2%	1%	1%	2%
PC34:2	2%	4%	1%	5%	1%
PC36:2	3%	2%	2%	2%	1%
PE34:1	10%	7%	4%	2%	6%
PE34:2	5%	5%	8%	3%	7%
PE36:1	7%	3%	3%	10%	11%
PE36:2	2%	1%	1%	2%	4%
SMd34:1	2%	4%	4%	3%	2%
SMd36:1	4%	3%	2%	3%	0%
SMd38:1	2%	5%	2%	2%	3%
SMd40:1	2%	3%	3%	4%	3%
SMd42:1	5%	7%	3%	6%	5%
